# Insulin Nanoemulsion Eye Drops for the Treatment of Dry Eye Disease in Sjögren’s Disease: A Randomized Clinical Trial Phase I/II

**DOI:** 10.3390/vision9030054

**Published:** 2025-07-09

**Authors:** Mateus Maia Marzola, Diego Rocha Gutierrez, Beatriz Carneiro Cintra, Adriana de Andrade Batista Murashima, Luciana Facco Dalmolin, Denny Marcos Garcia, Renata Fonseca Vianna Lopez, Fabiola Reis Oliveira, Eduardo Melani Rocha

**Affiliations:** 1 Core of Research in Ocular Physiopathology and Therapeutics (NAP-FTO), Ribeirao Preto 14049-900, Brazil; 2 Ribeirao Preto Medical School, University of Sao Paulo, Ribeirao Preto 14049-900, Brazil; 3 School of Pharmaceutical Sciences of Ribeirao Preto, University of São Paulo, Ribeirao Preto 14040-903, Brazil

**Keywords:** insulin nanoemulsion, dry eye, Sjögren’s disease, nanoemulsion eye drops, insulin eye drops, dry eye treatment, dry eye randomized clinical trial

## Abstract

Dry eye disease (DED) is a hallmark of primary Sjögren’s disease (SjD) and often resists conventional treatments like lubricant eye drops. Insulin nanoemulsions offer a potential solution by improving drug penetration and retention on the ocular surface. In animal models, insulin has shown benefits in promoting tear secretion and corneal healing. This study evaluated the safety and efficacy of insulin nanoemulsion eye drops (20 IU/mL, three times daily for 30 days) in patients with SjD. Thirty-two patients were randomized in a double-masked design to receive either insulin or placebo drops. Symptoms (assessed by OSDI questionnaire) and objective measures (tear film breakup time, corneal and conjunctival staining, and Schirmer Test) were recorded at baseline, after 4 weeks of treatment, and at a 4-week follow-up. Twenty-three participants completed the study. Both groups showed significant improvement in symptoms and objective signs after treatment (*p* < 0.05), but no significant differences were found between the insulin and placebo groups. No clinically relevant adverse effects were reported. Insulin nanoemulsion eye drops are safe for SjD patients, but their therapeutic advantage remains unclear. Further studies with larger samples, extended follow-up, and dose adjustments are needed to better understand their potential.

## 1. Introduction

Dry eye disease (DED) is a chronic condition with a multifactorial etiology that affects the ocular surface and tear film and may potentially lead to corneal and conjunctival damage. Sjögren’s disease (SjD), an autoimmune exocrinopathy, targets the lacrimal and salivary glands, resulting in diminished tear and saliva production, and is recognized as a cause of moderate to severe DED [1,2,3,4].

Based on reduced tear production and the inflammatory process, treating DED involves using artificial tears, topical anti-inflammatories and corticosteroids, dietary, lifestyle, and environmental adjustments, and surgical interventions in some critical cases. Therefore, studies to explore new therapeutic modalities are justified, as the most common treatment prescribed for DED, ocular lubricants, alleviates symptoms but does not address the underlying cause nor provides benefits to all patients [5,6,7].

In recent decades, insulin receptors have been identified on the ocular surface and in the lacrimal gland, while insulin has been detected in tears. Insulin production in the lacrimal gland is locally and systemically regulated and has a homeostatic role in the gland and the ocular surface [8,9,10,11,12]. This enlightenment supports the hypothesis of a potential therapeutic action of insulin topically applied in pathological processes of the ocular surface and DED. Topical insulin ophthalmic solutions have been studied to evaluate their healing effects on corneal injuries in animal and human models [13,14,15]. However, the bioavailability of the insulin molecule on the ocular surface is low, which prompted the development of an insulin nanoemulsion formulation designed to enhance its local bioavailability [16,17,18]. In animal models, this formulation promoted exocrine secretion, cornea preservation, and reduced inflammation in response to chemical injury [16]. Hypothetically, the insulin eye drop formulation in a vehicle containing lipid nanoemulsions may represent a valuable strategy for treating moderate to severe DED.

The evidence supporting the use of topical insulin for treating ocular surface diseases remains limited. While some masked randomized clinical trials have investigated its effects on persistent epithelial defects, iatrogenic corneal epithelial defects, and dry eye disease in patients with diabetes, further research is needed to establish its efficacy and clinical applicability [19,20,21,22].

This study aimed to evaluate the safety and efficacy of an insulin nanoemulsion formulation in patients with DED through a double-masked, randomized clinical trial. Given the multifactorial nature of DED, and even the heterogeneity of manifestations of SjD, only women diagnosed with primary SjD were selected to homogenize the sample [2,23,24,25].

This article is a revised and expanded version of a paper entitled “Insulin nanoemulsion eye drops for the treatment of dry eye disease in Sjögren’s Disease: A randomized clinical trial phase I/II”, which was presented at the 10th International Conference on the Tear Film & Ocular Surface: Basic Science and Clinical Relevance, held in Venice, Italy, from 31 October to 2 November 2024 [26].

## 2. Materials and Methods

### 2.1. Study Design

It was a Phase I/II, single-center, randomized, double-masked, vehicle placebo-controlled clinical trial. The study assessed the safety and efficacy of the insulin nanoemulsion eye drop (INS) with a concentration of 0.7 mg/mL (20 IU/mL) in the treatment of dry eye in patients with primary SjD, according to the ACR/EULAR criteria [27]. The insulin concentration at 0.7 mg/mL is based on the best concentration described in a randomized clinical trial, which compared different concentrations in patients with epithelial defects [28,29,30].

This study was conducted by the Division of Ophthalmology, Department of Otolaryngology, Ophthalmology, and Head and Neck Surgery at the Hospital das Clínicas, Ribeirão Preto Medical School (HCFMRP), São Paulo, Brazil. The study protocol and design were approved by the National Research Ethics Commission (CONEP-4.407.674), which was conducted following the guidelines for confidentiality of medical records and the Declaration of Helsinki. All participants agreed to and signed informed consent forms before initiating the study or any procedures. The study was registered in the Brazilian Registry of Clinical Trials (ReBEC-RBR-7p43fkc).

After consent, all eligible patients were randomized and evaluated according to their order of arrival, dividing them into two groups: one group received INS, and the other received a placebo (PCB). The PCB group received a formulation without the active ingredient, containing only the vehicle. Researchers, support staff, patients, and sponsors were masked during the process. All participants received identical-looking bottles and storage materials. The treatment duration was 4 weeks. Patients were instructed to use the eye drops 3 times a day, one drop in each eye, and to continue using their usual lubricants/artificial tears, allowing at least 30 min between the study eye drop and the lubricant. Three visits were conducted: Visit 01 (baseline) for complete evaluation and randomization; Visit 02, 2 weeks/15 days ± 3; Visit 03, 4 weeks/30 days ± 7.

### 2.2. Insulin Eyedrops

The insulin nanoemulsion eye drop was developed as follows: Oil-in-water (O/W) INS nanoemulsions (Sigma-Aldrich, Darmstadt, Germany) were prepared using low-frequency ultrasound at 20 kHz (Vibra-Cell VCX500, Sonics & Materials Inc., Newtown, CT, USA). Briefly, the oil phase (5% (*w*/*w*) medium-chain triglycerides and 4% (*w*/*w*) egg phosphatidylcholine (Lipoid E80)) were weighed and heated in a water bath (70 °C) until entirely melted. In another beaker, the aqueous phase (0.1% (*w*/*v*) Poloxamer 188 solution and 0.02% (*w*/*v*) of a cationic surfactant), along with 4% (*w*/*w*) polysorbate 80, was also heated to 70 °C. The aqueous phase was then poured over the oil phase, and the mixture was subjected to vortex mixing for 5 min, followed by ultrasound at 50% amplitude, with a 1 s ON and 1 s OFF duty cycle for 5 min. The INS was incorporated after sonication at a concentration of 0.7 mg/mL, equivalent to 20 IU/mL (1 IU/drop). Blank formulations (without active ingredients) were prepared for the placebo group.

Both insulin-loaded and placebo nanoemulsions were characterized by dynamic light scattering to determine the mean droplet size, polydispersity index (PDI), and zeta potential, as well as osmolarity and pH. The insulin-loaded nanoemulsion had a mean droplet size of 35±1.3nm, PDI of 0.191±0.01, zeta potential of −7±4mV, pH 7.35±0.03, and osmolarity of 264±3.6mOsm/L. The placebo formulation (nanoemulsion without insulin) showed similar characteristics: droplet size of 37.5±1.2nm, PDI of 0.209±0.01, zeta potential of −6.5±3.1mV, pH 6.97±0.13, and osmolarity of 265±1.7mOsm/L. These results confirm that the addition of insulin did not significantly alter the physicochemical properties or colloidal stability of the system.

Although insulin remains in the aqueous phase due to its hydrophilic nature, nanoemulsions provide a structured environment distinct from simple aqueous solutions. The presence of non-ionic surfactants, phospholipids, and a cationic co-surfactant modifies interfacial characteristics, favoring protein dispersion, protecting against enzymatic degradation, and enhancing residence time on the ocular surface.

The eye drops need to be stored at 4 °C, so volunteers were instructed to keep them refrigerated. A thermal container with reusable ice packs was provided to maintain the temperature during transport.

### 2.3. Patients

The inclusion criteria for volunteers were adults ≥ 18 years old, with a diagnosis of Sjögren’s disease according to the ACR-EULAR 2016 criteria and dry eye disease indicated by continuous use of artificial tears for the last four months, with Ocular Surface Disease Index (OSDI) questionnaire score ≥ 10 and basal tearing ≤ 5 mm on the Schirmer I Test (ST), or corneal fluorescein staining ≥3, or conjunctival Lissamine Green staining ≥3 in at least one eye [27,31].

Patients with the following conditions were excluded: active ocular infectious diseases, ocular inflammatory diseases not related to dry eye, those undergoing chemotherapy or radiotherapy, those using topical ocular medications (such as cyclosporine and antiglaucoma drugs), except for lubricating eye drops and ointments, and those who disagreed with or could not understand the terms of the informed consent.

### 2.4. Outcome Measures

All adverse events and treatment-emergent adverse events (TEAEs) were recorded for safety monitoring. Intraocular pressure (IOP) was measured throughout the study to detect any potential changes caused by the eye drops. Additionally, questionnaires were used to assess local and systemic symptoms. Dry eye signs and symptoms were evaluated at three time points: before treatment, 15 days after starting eye drops, and at the end of 30 days. The primary efficacy outcome was tear production, measured using the Schirmer I Test (without anesthesia). A 5 × 60 mm filter paper strip was placed in the lower conjunctival sac for 5 min, and the length of wetting (in millimeters) was recorded. These values were then compared across groups [31].

The secondary outcomes were changes in the Ocular Surface Disease Index (OSDI) questionnaire; the Tear Breakup Time (TBUT) test measured in seconds; corneal staining with fluorescein (CF); and conjunctival staining with 1% Lissamine Green (LG).

The secondary outcomes included changes in the Ocular Surface Disease Index (OSDI) questionnaire, which is validated in Portuguese and scored from 0 to 100, with the highest value indicating severe symptoms; the Tear Breakup Time (TBUT) was measured in seconds after fluorescein instillation into the conjunctival sac, instructing the volunteer to blink and then keep their eyes open for film breakup analysis under a slit lamp with a cobalt blue filter; the corneal staining with fluorescein (CF) was examined under a slit lamp with a blue filter, with changes graded from 0 to 15; the conjunctival staining with 1% Lissamine Green (LG) was assessed after instilling one drop of 1% lLissamine Green eye drop into the conjunctival sac, followed by biomicroscopic examination, with findings graded from 0 to 18 [31,32].

Adherence was assessed by the difference in weight between the eye drop bottles delivered and those returned, allowing for an estimate of the number of drops used during the period. Adherence was considered good if the application was between 80% and 120% of the estimated number of drops for the period.

### 2.5. Statistical Methods

The normality of the data was assessed using the Shapiro–Wilk test. The results are presented as mean and standard deviation (SD) for continuous and parametric variables or as median and interquartile range (IQR) otherwise. IOP was analyzed using a two-way ANOVA, and symptoms were analyzed using the Fisher test.

A linear mixed-effects model was used to analyze the ST, TBUT, OSDI, CF, and LG variables, accounting for fixed and random effects. Fixed effects included group and time, while random effects modeled the variability between subjects. The model appropriately accommodated the repeated structure of the data. Model assumptions, including normality and homoscedasticity of residuals, were verified using diagnostic plots. The significance of the fixed effects was assessed using Type III tests. When necessary, pairwise comparisons were conducted using Tukey’s adjustment for multiple testing. Log transformation was applied as needed.

Results were reported as estimated marginal means with 95% confidence intervals (CIs). The eye with the lower tear production on the Schirmer Test (ST) was used to evaluate and compare the results. The right eye was selected if tear production was similar between both eyes. All analyses were performed using R software version 4.1.1 (R Foundation for Statistical Computing, Vienna, Austria). A significance threshold of α = 0.05 was applied for all statistical tests.

## 3. Results

### 3.1. Follow-Up

**Figure 1:**
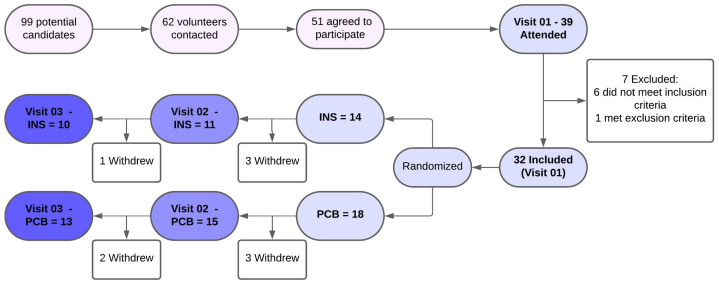
Flowchart of volunteers’ recruitment and enrollment. Abbreviations: INS: Insulin group, PCB: Placebo group.

Ninety-nine patients with Sjögren’s disease (SjD) from Hospital das Clínicas, Ribeirão Preto Medical School, were selected as potential candidates. Through phone contact, 62 patients were invited to the study; 51 agreed to attend the first visit, and 39 showed up. Of these, 32 patients were included; 6 were excluded for not meeting the inclusion criteria, and 1 was excluded for meeting the exclusion criteria. The study was conducted over three recruitment rounds. The included volunteers were randomly evaluated and randomized, based on the order of arrival, to receive either INS or PCB. The loss of participants during the follow-up was similar between the groups, with four cases in the INS group and five in the PCB group, all due to reasons unrelated to the study, such as transportation difficulties, family illness, work commitments, and infections (Dengue, COVID-19, and H1N1). Initially, 14 individuals were randomized into the INS group and 18 into the PCB group. By the end of the study, 23 patients completed it, with 10 volunteers in the INS group and 13 in the PCB group (Figure 1).

### 3.2. Overall Analysis

A total of 23 volunteers fully participated in the study and were randomly assigned to receive either the vehicle-containing eye drop (PCB, n = 13) or INS 20 IU/mL (n = 10). The groups’ demographic and clinical characteristics were comparable (*p* > 0.05). Also, the initial ocular measurements (Visit 01) related to signs and symptoms of DED and intraocular pressure (IOP) were statistically similar in both groups (Table 1).

### 3.3. Safety Results

No adverse effects resulting in death or preventing the use of eye drops have been reported. There was no statistically significant difference in intraocular pressure (IOP) among the volunteers evaluated throughout the study in both groups (*p*-value > 0.05). Local and systemic side effects were assessed using a structured anamnesis, with visual blurring being the only side effect that differed between the groups (*p* < 0.05), predominantly in the INS group, of mild intensity and limited to the period immediately following the application of the eye drops (Table 2).

### 3.4. Efficacy Results

The analysis of the Schirmer I Test (ST), Ocular Surface Disease Index (OSDI) score, corneal staining with fluorescein (CF), conjunctival staining with 1% Lissamine Green (LG), and the TBUT test revealed no statistically significant differences were found between the treatment groups (*p* > 0.05). A significant temporal effect was observed in the OSDI and LG scores (*p* < 0.05), while CF demonstrated a statistically significant group-time interaction (*p* < 0.05) (Table 3).

### 3.5. Volunteers’ Adherence to the Protocol

In addition to questioning participants at each follow-up visit and allowing them to report discomfort or discontinue treatment at any time, adherence to the protocol was assessed by weighing the eye drop bottles before and after the treatment period. By comparing the weight of the bottles dispensed and returned, we estimated adherence for each group. Adherence was considered acceptable if the bottle usage fell between 80% (minimum expected consumption) and 120% (maximum tolerated consumption) of the liquid’s weight over the treatment cycle. We observed that the INS group (n = 10) showed better adherence to the study, with 70.0% of the volunteers demonstrating appropriate usage (between 80% and 120% of the expected amount). However, one volunteer was considered non-adherent and did not return the eye drop bottle for weighing. The PCB group (n = 13) showed an adherence rate of 15.38% throughout the study, with a statistically significant difference between the groups (*p* < 0.05).

## 4. Discussion

The use of insulin in treating ocular surface diseases is not new [33]. Curiously, despite the several direct and experimental evidence of its therapeutic benefits and the lack of an effective approach to promote corneal wound healing and minimize the damage of moderate to severe DED, the strategy of using insulin therapy for ocular surface diseases was not recalled until recently.

To the best of our knowledge, this is the first double-masked, randomized clinical trial investigating the use of a topical insulin nanoemulsion for DED in SjD. Additionally, lipid nanodispersion technology was intentionally incorporated as a vehicle to enhance the bioavailability of the therapeutic molecule on the ocular surface [16,28,29,30].

Besides several recent studies with different insulin eye drops, as mentioned before, insulin topical treatment with nanotechnology delivery for skin wound healing is also attracting the attention of researchers. Moreover, recent trials revealed that insulin eye drops improved DED symptoms after a short-term application in different doses and specific diseases [28,29,30].

The supports for this strategy are the pro-mitotic, antioxidant, and anti-inflammatory effects of insulin, the nanoemulsion formulation that increases the stability and delivery of the active principle, and the local application that prevents systemic side effects [11,12,16,17].

Some aspects of the topical insulin applicability remain under discussion, such as the insulin subtype, concentration, daily usage frequency, and treatment duration. We chose a 1 IU/drop dose (20 IU/mL) based on the best concentration described in a randomized clinical trial, which compared different concentrations in patients with epithelial defects [28]. Although our study recommended storing the eye drops at 4 °C, two recent studies showed that topical insulin remains stable at room temperature for 90 days, facilitating its use [34,35].

The comparisons of OSDI score, TBUT, keratitis (CF), Lissamine Green staining, tear secretion (ST), and IOP showed a positive trend favoring the insulin eye drops (INS) but without statistical significance. The difficulty in reaching a large sample size due to strict diagnostic criteria and sample homogeneity, test variability within groups, short follow-up period, and poor adherence to treatment, with dropout rates and non-adherence to the prescribed treatment in 40% of participants, highlights the challenges in conducting reasonable therapeutic clinical studies.

On the other hand, the lack of deterioration in ocular exam parameters and the comfort reported by individuals who completed the study suggest that the insulin nanoemulsion eye drops at a concentration of 20 IU/mL are safe [36,37]. Future studies will aim to assess the effect of this medication at different doses and regimens in other ocular conditions, using more sensitive methods for monitoring therapeutic efficacy.

Despite being one of the most common conditions bringing patients to eye clinics, dry eye disease (DED) has no definitive treatment, and managing the condition is fraught with several challenges [7,38,39]. These encompass various pathophysiological processes, symptoms that do not always align with objective examination findings, differing stages of ocular surface disease, and medications with limited effectiveness. All factors, coupled with the limitations of diagnostic methods, make conducting clinical trials difficult.

Even so, several studies have demonstrated that nanoemulsions can efficiently incorporate and deliver therapeutic proteins even when added post-emulsification, provided that suitable surfactant systems are used [40,41]. Moreover, nanoemulsions act as both drug carriers and tear film stabilizers, particularly by restoring its lipid layer [42], which is compromised in dry eye disease. This dual functionality may help explain the absence of significant differences between treatment and placebo groups, emphasizing the intrinsic therapeutic potential of the nanoemulsion itself.

The strengths of this study include focusing on a single causative condition of DED, SjD, randomly assigning patients to treatment or placebo groups (with the placebo group receiving the formulation vehicle), and evaluating both clinical outcomes and patient adherence to the protocol. While adherence monitoring was a key aspect of the study, lower adherence was observed in the placebo group, highlighting the challenges of treatment compliance in DED research. Given that adherence is a crucial factor in therapeutic success, it is often underestimated in clinical practice, clinical trials, and studies on DED [37,38].

The weak points of our work are the limited size of the initial sample, attributed to SjD individuals’ reluctance to visit hospitals during and right after the pandemic. Additionally, about 20% of SjD individuals did not complete the protocol for different reasons. Furthermore, what could sound like a contradictory statement compared to the strengths of the study, but is revealing, is the surprising levels of low adherence to the clinical protocol.

So far, clinical trials, including this one, have not demonstrated the efficacy of insulin eye drops for treating DED. Therefore, our findings do not support the widespread off-label use of this hormone, which has been observed in different countries at varying doses and formulations. We hope that this study will help guide future research by testing different insulin dosages, larger patient groups, more severe DED cases, other DED subtypes, longer follow-up periods, and more sensitive tools to assess clinical variations in the ocular surface and tear film, ultimately expanding knowledge on this potential therapy.

In conclusion, the lack of deterioration in the ocular parameters and the comfort reported by those who completed the study suggest that the 20 IU/mL insulin nanoemulsion eye drops are safe to treat DED in SjD (Table 2 and Table 3). However, the study design and some methodological pitfalls limited the statement about its efficacy.

## 5. Patents

The authors, Eduardo Melani Rocha and Renata Fonseca Vianna Lopez, hold the rights to the patents for the insulin nanoemulsion eye drops regarded by the Instituto Nacional da Propriedade Intelectual (BR 102015005856-0).

## Figures and Tables

**Table 1:** Demographic and baseline clinical characteristics of SjD individuals that concluded the insulin eye drops clinical trial phase I/II.

	No	
**Characteristcs**	**Insulin 20 IU/mL (n = 10)**	**Placebo (n = 13)**
Age, mean (SD) y	55.30 (14.84)	59.15 (13.28)
Female, No. (%)	10 (100)	13 (100)
Race or ethnicity, No. (%)		
White	7 (70)	9 (69.23)
Black	3 (30)	4 (30.77)
Duration of Sjd, means (SD) y	8.80 (5.51)	12.17 (10.69)
IOP, mean (SD), mmHg	Visit 01 (n = 4)	Visit 01 (n = 9)
	OD: 13.50 (3.42)	OD: 13.67 (2.60)
	OS: 14.00 (3.37)	OS: 13.33 (2.00)

Abbreviations: IOP: intraocular pressure, OD: right eyes; OS: left eye, SD: standard deviation.

**Table 2:** Signs and symptoms reported and mean intraocular pressure (IOP) measured throughout the study.

		Insulin			
**Index**		**20 IU/mL**	**Placebo**		
		**(n = 4)**	**(n = 5)**		***p*** **-Value**
Headache	Visit 02	1	0		>0.05
	Visit 03	0	1		>0.05
Dizziness	Visit 02	0	1		>0.05
	Visit 03	0	1		>0.05
Insomnia	Visit 02	0	0		>0.05
	Visit 03	0	0		>0.05
Appetite disturbance	Visit 02	0	0		>0.05
	Visit 03	0	1		>0.05
Ocular pruritus	Visit 02	3	2		>0.05
	Visit 03	3	3		>0.05
Ocular burning	Visit 02	2	1		>0.05
	Visit 03	3	2		>0.05
Visual blurring	Visit 02	3	1		>0.05
	Visit 03	4	1		0.046 *
Dry eye	Visit 02	0	0		>0.05
	Visit 03	0	0		>0.05
Others *	Visit 02	0	1		>0.05
	Visit 03	1	0		>0.05
IOP, mean (SD), mmHg		OD: 12.25 (4.50)		OD: 11.67 (2.12)	>0.05
	Visit 02	OS: 12.50 (4.72)	(n = 9)	OS: 11.11 (2.52)	
	Visit 03	OD: 13.75 (5.56)	(n = 9)	OD: 13.78 (1.92)	>0.05
		OS: 13.25 (4.99)		OS: 12.67 (1.80)	

Abbreviations: IOP: intraocular pressure, OD: right eyes; OS: left eye, SD: standard deviation. * Palpebral burning.

**Table 3:** Changes in dry eye signs and symptoms during the insulin nanoemulsion eye drop Phase I/II clinical trial.

		Insulin 20 IU/mL (n = 10)		Placebo (n = 13)		Mixed Model (*p*-Value)		
**Index**	**Time Point**	**Value (LSM)**	**95% CI**	**Value (LSM)**	**95% CI**	**Group**	**Time**	**Group × Time**
ST (*), mm	Visit 01	3.8	1.1–9.9	3.3	1.1–7.8			
	Visit 02	4.6	1.5–11.6	5.8	2.3–13.0			
	Visit 03	4.5	1.4–11.3	4.0	1.4–9.1	0.993	0.242	0.590
OSDI score	Visit 01 ^a,b^	47.2	31.4–63.0	64.8	51.0–78.7			
	Visit 02 ^a^	26.9	11.1–42.7	37.2	23.4–51.1			
	Visit 03 ^b^	29.5	13.7–45.3	27.1	13.2–40.9	0.261	0.0002	0.301
CF score	Visit 01	3.8	1.2–6.4	6.5	4.2–8.7			
	Visit 02	5.2	2.6–7.8	4.8	2.5–7.0			
	Visit 03	4.9	2.3–7.5	5.4	3.1–7.6	0.568	0.933	0.0069
LG score	Visit 01	5.4	3.0–7.8	8.4 ^c^	6.3–10.5			
	Visit 02	3.7	1.3–6.1	3.2 ^c^	1.1–5.3			
	Visit 03	5.0	2.6–7.4	5.7	3.6–7.8	0.407	0.0006	0.1074
TBUT test (*), s	Visit 01	6.1	3.1–11.1	3.5	1.8–2.0			
	Visit 02	3.1	1.4–6.1	3.1	1.6–5.6			
	Visit 03	3.0	1.3–5.8	3.1	1.6–5.6	0.643	0.075	0.221

Abbreviations: LSM: Least Square Mean, ST: Schirmer Test, OSDI: Ocular Surface Disease Index, CF: corneal staining with fluorescein, LG: corneal staining with 1% Lissamine Green, TBUT: Tear Breakup Time test. The superscript letters (^a^, ^b^, ^c^) indicate differences between the time in the Tukey HSD post hoc. The asterisks (*) represent the tests in which the values were log-transformed, and the geometric mean was then calculated. The underline sign indicate statistically significant changes.

## Data Availability

The original contributions presented in this study are included in this article. Further inquiries can be directed to the corresponding authors.

## Abbreviations

The following abbreviations are used in this manuscript:

DED	Dry eye disease
SjD	Sjögren’s disease
IU	International unit
INS	Insulin
PCB	Placebo
OSDI	Ocular Surface Disease Index
ST	Schirmer Test
TEAEs	Treatment-emergent adverse events
IOP	Intraocular pressure
TBUT	Tear Breakup Time
CF	Corneal staining with fluorescein
LG	Conjunctival staning with 1% Lissamine Green
SD	Standard Deviation
IQR	Interquartile range
CI	Confidence Interval

## References

[1] Craig J.P., Nichols K.K., Akpek E.K., Caffery B., Dua H.S., Joo C.K., Liu Z., Nelson J.D., Nichols J.J., Tsubota K. (2017). TFOS DEWS II Definition and Classification Report. Ocul. Surf..

[2] Stapleton F., Alves M., Bunya V.Y., Jalbert I., Lekhanont K., Malet F., Na K.-S., Schaumberg D., Uchino M., Vehof J. (2017). TFOS DEWS II Epidemiology Report. Ocul. Surf..

[3] Rouen P.A., White M.L. (2018). Dry Eye Disease: Prevalence, Assessment, and Management. Home Healthcare Now.

[4] Parisis D., Chivasso C., Perret J., Soyfoo M.S., Delporte C. (2020). Current State of Knowledge on Primary Sjogren’s Syndrome, an Autoimmune Exocrinopathy. J. Clin. Med..

[5] Jones L., Downie L.E., Korb D., Benitez-del-Castillo J.M., Dana R., Deng S.X., Dong P.N., Geerling G., Hida R.Y., Liu Y. (2017). TFOS DEWS II Management and Therapy Report. Ocul. Surf..

[6] Kim M., Lee Y., Mehra D., Sabater A.L., Galor A. (2021). Dry eye: Why artificial tears are not always the answer. BMJ Open Ophthalmol..

[7] Alves M., Fonseca E.C., Alves M.F., Malki L.T., Arruda G.V., Reinach P.S., Rocha E.M. (2013). Dry eye disease treatment: A systematic review of published trials and a critical appraisal of therapeutic strategies. Ocul. Surf..

[8] Rocha E.M., Cunha D.A., Carneiro E.M., Boschero A.C., Saad M.J., Velloso L.A. (2002). Identification of insulin in the tear film and insulin receptor and IGF-1 receptor on the human ocular surface. Investig. Ophthalmol. Vis. Sci..

[9] Cunha D.A., Carneiro E.M., Alves M.d.C., Jorge A.G., de Sousa S.M., Boschero A.C., Saad M.J., Velloso L.A., Rocha E.M. (2005). Insulin secretion by rat lacrimal glands: Effects of systemic and local variables. Am. J. Physiol.-Endocrinol. Metab..

[10] Cunha D.A., Alves M.C., Stoppiglia L.F., Jorge A.G., Módulo C.M., Carneiro E.M., Boschero A.C., Saad M.J., Velloso L.A., Rocha E.M. (2007). Extra-pancreatic insulin production in rat lachrymal gland after streptozotocin-induced islet beta-cells destruction. Biochim. Biophys. Acta.

[11] Módulo C.M., Jorge A.G., Dias A.C., Braz A.M., Bertazolli-Filho R., Jordão A.A., Marchini S.J., Rocha E.M. (2009). Influence of insulin treatment on the lacrimal gland and ocular surface of diabetic rats. Endocrine.

[12] Dias A.C., Batista T.M., Roma L.P., Módulo C.M., Malki L.T., Dias L.C., Alves M., Reinach P.S., Carneiro E.M., Rocha E.M. (2015). Insulin replacement restores the vesicular secretory apparatus in the diabetic rat lacrimal gland. Arq. Bras. Oftalmol..

[13] Zagon I.S., Sassani J.W., McLaughlin P.J. (2006). Insulin treatment ameliorates impaired corneal reepithelialization in diabetic rats. Diabetes.

[14] Zagon I.S., Klocek M.S., Sassani J.W., McLaughlin P.J. (2007). Use of topical insulin to normalize corneal epithelial healing in diabetes mellitus. Arch. Ophthalmol..

[15] Bastion M., Ling K.P. (2013). Topical insulin for healing of diabetic epithelial defects?: A retrospective review of corneal debridement during vitreoretinal surgery in Malaysian patients. Med. J. Malays..

[16] Cruz-Cazarim E.L.C., Cazarim M.S., Ogunjimi A.T., Petrilli R., Rocha E.M., Lopez R.F.V. (2019). Prospective insulin-based ophthalmic delivery systems for the treatment of dry eye syndrome and corneal injuries. Eur. J. Pharm. Biopharm..

[17] Sullivan D.A., Rocha E.M., Aragona P., Clayton J.A., Ding J., Golebiowski B., Hampel U., McDermott A.M., Schaumberg D.A., Srinivasan S. (2017). TFOS DEWS II Sex, Gender, and Hormones Report. Ocul. Surf..

[18] Gaudana R., Jwala J., Boddu S.H., Mitra A.K. (2009). Recent perspectives in ocular drug delivery. Pharm. Res..

[19] Fai S., Ahem A., Mustapha M., Noh U.K.M. (2017). Randomized Controlled Trial of Topical Insulin for Healing Corneal Epithelial Defects Induced During Vitreoretinal Surgery in Diabetics. Asia-Pac. J. Ophthalmol..

[20] Dasrilsyah A.M., Halim W.H.W.A., Mustapha M., Tang S.F., Kaur B., Ong E.Y., Bastion M.L.C. (2023). Randomized Clinical Trial of Topical Insulin Versus Artificial Tears for Healing Rates of Iatrogenic Corneal Epithelial Defects Induced During Vitreoretinal Surgery in Diabetics. Cornea.

[21] Azmi N.A., Bastion M.C. (2020). Short-term results of trial of topical insulin for treatment of dry eyes in diabetics. Eye Contact Lens.

[22] Tahmaz V., Menghesha L., Stern M.E., Holtick U., Scheid C., Steven P. (2024). Insulin eye drops for severe refractory chronic ocular graft-versus-host disease. Bone Marrow Transplant..

[23] Restrepo-Jimenez P., Molano-Gonzalez N., Anaya J.M. (2019). Geoepidemiology of Sjogren’s syndrome in Latin America. Jt. Bone Spine.

[24] Garcia D.M., Oliveira F.R., Módulo C.M., Faustino J., Barbosa A.P., Alves M., Rocha E.M. (2018). Is Sjögren’s syndrome dry eye similar to dry eye caused by other etiologies? Discriminating different diseases by dry eye tests. PLoS ONE.

[25] Oliveira F.R., Motta A.C.F., Módulo C.M., Garcia D.M., Chiorini J.A., Louzada-Junior P., Rocha E.M. (2022). Clinical and laboratory evaluation of sicca complaints: Distinctive aspects of primary, secondary and non-Sjogren syndrome. Adv. Rheumatol..

[26] Marzola M.M., Gutierrez D.R., Cintra B.C., Murashima A.d.A.B., Dalmolin L.F., Garcia D.M., Lopez R.F.V., de Oliveira F.R., Rocha E.M. Insulin nanoemulsion eye drops for the treatment of dry eye disease in Sjögren’s Disease: A randomized clinical trial phase I/II. Proceedings of the 10th International Conference on the Tear Film & Ocular Surface: Basic Science and Clinical Relevance.

[27] Shiboski C.H., Shiboski S.C., Seror R., Criswell L.A., Labetoulle M., Lietman T.M., Rasmussen A., Scofield H., Vitali C., Bowman S.J. (2017). 2016 American College of Rheumatology/European League Against Rheumatism classification criteria for primary Sjogren’s syndrome: A consensus and data-driven methodology involving three international patient cohorts. Ann. Rheum. Dis..

[28] Chauhan S., Jhawat V., Singh R.P., Yadav A. (2024). Topical delivery of insulin using novel organogel formulations: An approach for the management of diabetic wounds. Burns.

[29] Atikah A., Suzana M., Wan Haslina W.A.H., Norshamsiah M.D., Mushawiahti M., Birinder K.S.S., Tang S.F., Bastion M.L.C. (2024). Randomized controlled trial on effects of topical insulin compared to artificial tears and normal saline on tear inflammatory mediator levels and clinical parameters in diabetics with dry eye disease. Cont. Lens Anterior Eye.

[30] Quitério M., Simões S., Ascenso A., Carvalheiro M., Leandro A.P., Correia I., Viana A.S., Faísca P., Ascensão L., Molpeceres J. (2021). Development of a topical insulin polymeric nanoformulation for skin burn regeneration: An experimental approach. Int. J. Mol. Sci..

[31] Wolffsohn J.S., Arita R., Chalmers R., Djalilian A., Dogru M., Dumbleton K., Gupta P.K., Karpecki P., Lazreg S., Pult H. (2017). TFOS DEWS II Diagnostic Methodology report. Ocul. Surf..

[32] Prigol A.M., Tenório M.B., Matschinske R., Gehlen M.L., Skare T. (2013). Translation and validation of ocular surface disease index to Portuguese. Arq. Bras. Oftalmol..

[33] Aynsley T.R. (1945). The use of insulin in the treatment of corneal ulcers. Br. J. Ophthalmol..

[34] Cuartero-Martínez A., Hermelo-Vidal G., Castro-Balado A., Gómez-García Á., González-Barcia M., Otero-Espinar F.J., Fernández-Ferreiro A., Mondelo-García C. (2022). Stability of insulin eye drops in the treatment of refractory corneal ulcers. Farm. Hosp..

[35] Vicario-de-la-Torre M., Puebla-García V., Ybañez-García L., López-Cano J.J., González-Cela-Casamayor M.A., Brugnera M., Burgos-Blasco B., Díaz-Valle D., Gegúndez-Fernández J.A., Benítez-Del-Castillo J.M. (2024). Topical insulin eye drops: Stability and safety of two compounded formulations for treating persistent corneal epithelial defects. Pharmaceutics.

[36] Dana R., Bradley J.L., Guerin A., Pivneva I., Stillman I.Ö., Evans A.M., Schaumberg D.A. (2019). Estimated prevalence and incidence of dry eye disease based on coding analysis of a large, all-age United States health care system. Am. J. Ophthalmol..

[37] Wolffsohn J.S., Semp D.A., Dutta D., Jones L., Craig J.P. (2025). TFOS ambassadors. Clinical practice patterns in the management of dry eye disease: A TFOS international survey 2023–2024. Ocul. Surf..

[38] Anghel L.A., Farcas A.M., Oprean R.N. (2019). An overview of the common methods used to measure treatment adherence. Med. Pharm. Rep..

[39] Novack G.D., Asbell P., Barabino S., Bergamini M.V.W., Ciolino J.B., Foulks G.N., Goldstein M., Lemp M.A., Schrader S., Woods C. (2017). TFOS DEWS II Clinical Trial Design Report. Ocul. Surf..

[40] Tsai M.J., Fu Y.S., Lin Y.H., Huang Y.B., Wu P.C. (2014). The Effect of Nanoemulsion as a Carrier of Hydrophilic Compound for Transdermal Delivery. PLoS ONE.

[41] Ali F.R., Shoaib M.H., Ali S.A., Yousuf R.I., Siddiqui F., Raja R., Jamal H.S., Saleem M.T., Ahmed K., Imtiaz M.S. (2022). A nanoemulsion based transdermal delivery of insulin: Formulation development, optimization, In-Vitro permeation across Strat-M^®^ membrane and its pharmacokinetic/pharmacodynamic evaluation. J. Drug Deliv. Sci. Technol..

[42] Chatzidaki M.D., Mitsou E. (2025). Advancements in Nanoemulsion-Based Drug Delivery Across Different Administration Routes. Pharmaceutics.

